# Alveolar bone changes following bi-maxillary vertical molars’ movements using clear aligners

**DOI:** 10.1186/s12903-023-03028-5

**Published:** 2023-05-25

**Authors:** Leena Ali Al-Warafi, Naseem Ali Al-Worafi, Maged Ali Al-Aroomi, Najah Alhashimi, Majedh Abdo Ali Alsomairi, Remsh Khaled Al-Rokhami, Xi Chen, Maged Sultan Alhammadi

**Affiliations:** 1 Department of Stomatology, The First Affiliated Hospital of Xi’an JiaotongUniversity, Xi’an, 710061 Shaanxi People’s Republic of China; 2 Department of Orthodontics, School of Stomatology, China Medical University, Shenyang, China; 3 Department of Oral and maxillofacial surgery, Faculty of dentistry, IBB University, IBB, Yemen; 4 College of Dental Medicine, Chief of orthodontics division at Hamad Medical Corporation, Qatar University, Doha, Qatar; 5 Division of Orthodontics and Dentofacial Orthopedics, Department of Preventive Dental Sciences, College of Dentistry, Jazan University, Jazan, Saudi Arabia

**Keywords:** Alveolar bone, Clear aligners, Cone Beam Computed Tomography (CBCT), Maxillary and mandibular molars, Vertical movements

## Abstract

**Objective:**

This study aimed to assess the alveolar bone changes following maxillary and mandibular molars’ intrusion and extrusion movements using Clear Aligners using Cone-Beam Computed Tomography (CBCT).

**Materials and methods:**

This is a retrospective clinical study in which 24 adult patients with pre-designed selection criteria and a mean age of 31.1 + 9.9 years were enrolled. The alveolar bone changes around one hundred thirty-three maxillary and mandibular molars intruded or extruded by Clear Aligners therapy were analyzed from CBCT using Invivo 6.0 software. Intra- and inter-examiner reliability analysis was performed using the intra-class correlation coefficient (ICC) and Cronbach’s Alpha statistics. The paired t-test was used to analyze significant differences before and after treatment (T0-T1). The significance level was considered at P < 0.05.

**Result:**

The patients were divided into two groups: extrusion (48.9%, n = 65 molars’ root) and intrusion (51.1%, n = 68 molars’ root) group. There was a significant decrease in the alveolar bone changes in the buccal surface of the mandibular right and left 1st molars in the extrusion group (-1.05 ± 0.97, -0.76 ± 1.12 mm, respectively) and the maxillary left 2nd molars in intrusion group (-0.42 ± 0.77 mm), and the lingual surface of intrusion of the mandibular left 1st molar (-0.64 ± 0.76 mm). Comparing the mean maxillary and mandibular changes (T0-T1) of both studied groups showed that the buccal alveolar bone changes for the left 1st and right 2nd molars showed a significant difference in extrusion and intrusion groups, respectively.

**Conclusions:**

The buccal alveolar bone changes is considered the most affected surface following maxillary and mandibular molars’ intrusion and extrusion movements using clear aligners, with mandibular molars being more affected than the maxillary ones.

## Introduction

The benefit/risk ratio is always considered during orthodontic treatment planning; maintaining and/or even achieving healthy supporting alveolar bone and periodontal ligament with the least undesirable iatrogenic effects are considered the primary aim of orthodontic treatment [1]. The successful treatment of malocclusion depends on the reaction of surrounding bone tissue; the orthodontist must be familiar with its physical properties, histology, and normal anatomy with normal variations [2]. The effect of orthodontic movement will depend on the applied force’s direction, magnitude, and duration [3]. Orthodontic treatment could influence alveolar bone changes and the longer the treatment, the more significant loss of the crest of the alveolar bone [4–6].

Bone loss alters the center of resistance of the teeth, and consequently, conventional orthodontic treatments may be at significant risk because this morphological change will increase the moment of force with all consequences [7]. Besides, vertical tooth movements such as intrusion or extrusion may affect the distance between the cementoenamel junction (CEJ) and the crest of the alveolar bone after orthodontic treatment [7]. Therefore, verifying the true capability for bone remodeling in the alveolar bone is crucial to avoid unwanted side effects.

Orthodontic treatment can be performed mainly by either removable or fixed orthodontic appliances. With the recent increase in adults seeking orthodontic treatment, there has been a proportional increase in the need for orthodontic appliances that are both more comfortable and more esthetic than classical fixed orthodontic appliances. Clear aligners fulfilled these criteria; it depends on applying optimal orthodontic force to move teeth smoothly using others as anchorage unit/s [8]. Although clear aligners are professional in performing vertical tooth movement, it is complicated, if not impossible, to extrude molars teeth without attachments effectively. It is hard to extrude the teeth with a clear aligner due to loss of retention for extrusion [8]. While intruded molars teeth do not need any attachments because the occlusal surfaces adequately deliver the axial load, anchorage teeth may have an undesirable extrusion. Again, attachments on the premolars can be conventional for retention or optimized for extrusion and retention [9, 10].

Previous studies have concentrated on changes in the alveolar bone before and after conventional orthodontic treatment using bitewing or periapical radiographs [11, 12]. However, owing to the limitations of two-dimensional images, technical shortcomings such as magnification, geometric distortion, and overlap of structures restricted the reliability of their results [13]. Moreover, few studies have published their results regarding using Cone Beam Computed Tomography (CBCT) to evaluate changes in the alveolar bone level after incisor intrusion tooth movements using conventional orthodontic appliances [1, 14]. However, it appears that searching the available literature showed that no published study has reported evaluating the alveolar bone changes following maxillary and mandibular molars’ intrusion and extrusion movements using clear aligners using Cone-Beam Computed Tomography.

The null hypothesis is that there is no difference in the alveolar bone changes following maxillary and mandibular molars’ intrusion and extrusion movements following clear aligners. Therefore, this study aimed to assess the alveolar bone changes following maxillary and mandibular molars’ intrusion and extrusion movements following clear aligners therapy using Cone-Beam Computed Tomography (CBCT).

## Materials and methods

### Patients’ selection

This retrospective clinical study was approved by the ethics committee of the Department of Stomatology, First Affiliated Hospital, College of Medicine, Xi’an Jiaotong University, China (XJTU1AF2022LSK-186). All patients were requested to sign an electronic written informed consent for the treatment with clear aligners prior to orthodontic treatment.

### Sample size

The sample size was calculated using G* Power software (v3.1.3; Franz Faul, Universität Kiel, Germany) with an alpha value of 0.05 and a power of 95% based on the study conducted by Zhou et al. [15] in which the alveolar bone changes in the labial surface of the maxillary incisors was 0.73 ± 0.34 mm and 1.30 ± 0.6 mm in pre- and post-treatment, respectively. The resulting sample size was a minimum of 11 patients to be included in the study. This number was increased to a minimum of 12 patients in each studied group.

### Selection criteria

All patients treated with clear aligners were screened between January 2017 and December 2020 in the Department of Stomatology, Xi’an Jiaotong University, China. The inclusion criteria included: (1) Age ranged between 16 and 48 years; (2) Classified as skeletal Class II, > 4.7^o^ < 0.7^o^ malocclusion based on the ethnic group ANB angle norms; [16] (3) Sufficient space for molar distalization without the use of temporary anchorage devices; (4) First and second molars should be present; (5) Complete root formation; (6) Mild to moderately crowded arches. The exclusion criteria were: (1) Root caries or fractures; (2) Periodontal or gingival problems at the beginning of treatment; (3) Extraction treatment; (4) History of craniofacial syndromes or bone diseases; (5) Medications affected normal bone turnover; and (6) Low-quality CBCT before and/or after clear aligners treatment. The patients were divided according to the type of orthodontic movement into intrusion and extrusion groups.

### Alveolar bone changes assessment

Three-dimensional images were acquired using the CBCT machine (KaVo Company, Germany). The imaging parameters were set at 120 kV, 5 mA, the field of view (23 cm × 17 cm), and 17.8s exposure time, with a voxel size of 0.3 mm and a slice thickness of 2 mm. The patient was positioned upright and closed the teeth to their maximum intercuspation with the Frankfort horizontal plane parallel to the floor and the midsagittal plane perpendicular to the floor; all patients were instructed not to swallow during scanning. The collected CBCT scan before treatment (T0) and after treatment (T1) were transferred into DICOM (Digital Imaging and Communication in Medicine) file format and afterward imported into Invivo 6.0 software (Anatomage, San Jose, CA, USA). Using section view features (multiplanar view), which possess the ability of 3D view the X, Y, and Z sections (axial, coronal, and sagittal, respectively).

The sagittal, axial, and coronal views were properly oriented to measure each molar tooth’s buccal and lingual alveolar bone changes. Orientation of the axial view of the dental arch was done so that the long axis of the intended tooth is perpendicular to both coronal and sagittal coordinates (Fig. 11-A). Adjustment of the axial coordinate so that it crosses the other coordinate at the center of the intended tooth (Fig. 11-B and C) [17–19].

**Fig 1:**
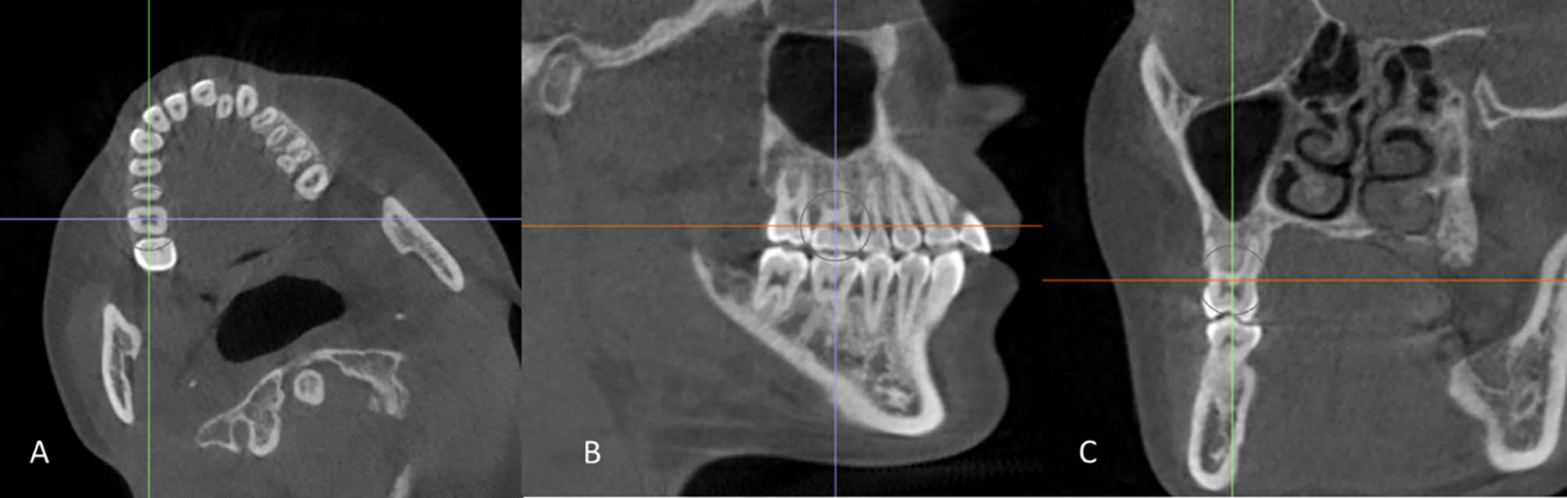
Steps of CBCT orientation with intersection of the coronal (blue), and axial (red) planes; **A** Orientation of the sagittal view of the dental arch was done so that the long axis of the intended tooth is perpendicular to both coronal and sagittal coordinate. **B** and **C**, Adjustment of the axial coordinate so that it crosses the other coordinate at the center of the intended tooth

**Fig 2:**
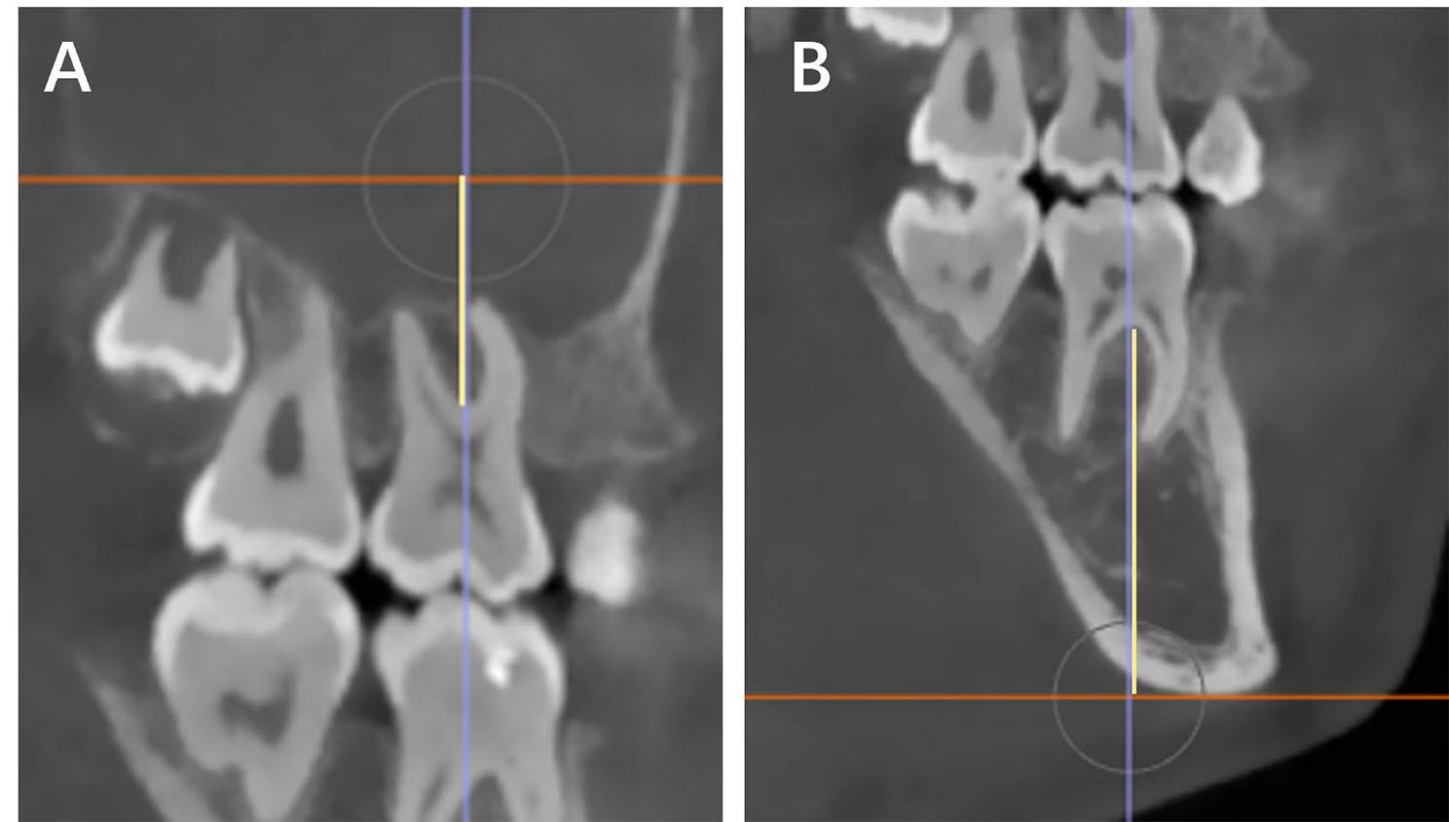
Molars’ intrusion and extrusion measurements. **A)**, the red line represents the palatal plane (ANS to PNS): and the yellow line represents the intersection line between the palatal plane and the most superior bi-furcation point of maxillary molars’ teeth. **B)**, the red line represents the mandibular plane (Me-Go): and the yellow line represents the intersection line between the mandibular plane and the most inferior bi-furcation point of mandibular molars’ teeth

To measure intrusion and extrusion of molars teeth in the axial view, after proper orientation of the slices, two reference planes were utilized in the sagittal view: for maxillary molars, the palatal plane (constructed by projecting a line through Anterior Nasal Spine, ANS, and Posterior Nasal Spine, PNS) was used, and for the mandibular molars the mandibular plane (constructed by projecting a line between the Gonion, Go, and menton, Me points) was used. The millimetric perpendicular distance between the most inferior point of the tri-furcation (Fig. 2, A) and the bi-furcation (Fig. 2, B) points of the maxillary and mandibular molars relative to the opposing planes were recorded, respectively. The difference between the pre-treatment and post-treatment was calculated.

**Fig 3:**
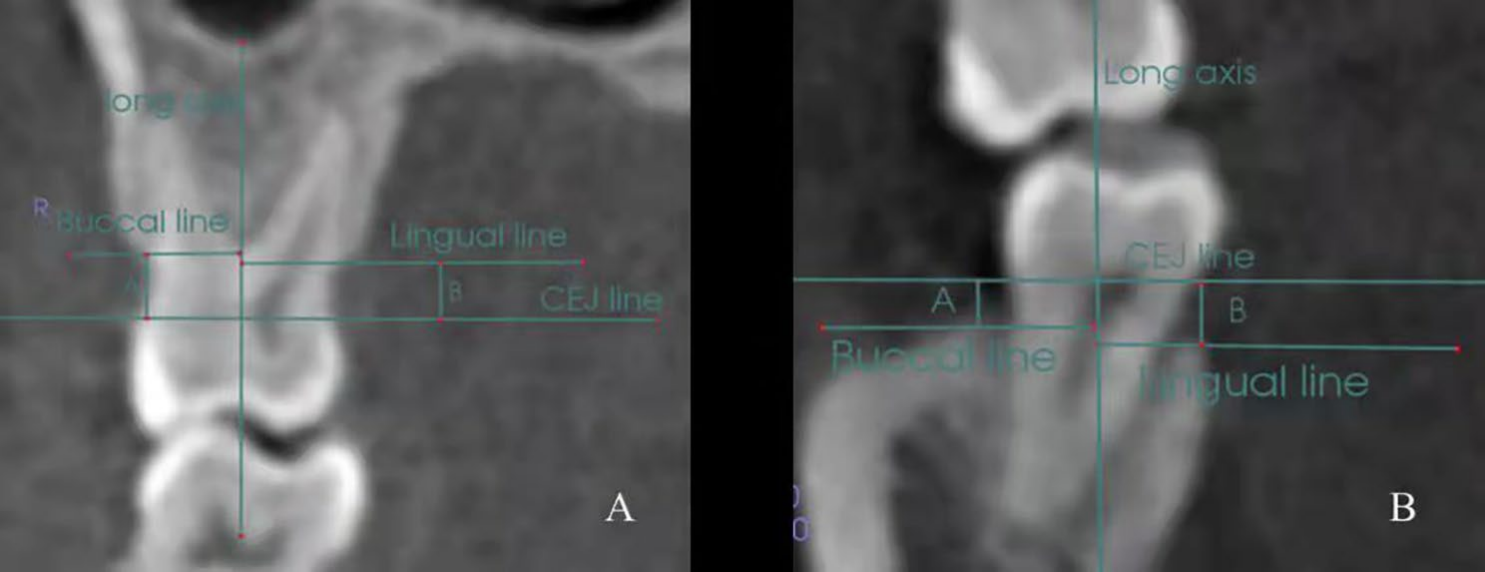
The reference lines for measurement the CABH: **A**, maxillary 1st molar; **B**, mandibular 2nd molar. **A, B:** The distances between CEJ line and crest of alveolar bone lines on the buccal and palatal/lingual surfaces

Following the precise orientation of the three planes, in coronal view, three reference lines parallel to each other and perpendicular to the long axis of the tooth were drawn. The first line from CEJ of the buccal surface to CEJ of the palatal/lingual surface; the second line from the crest of alveolar bone of the buccal surface to the long axis of the tooth; the third line from the crest of alveolar bone (CAB) of the palatal/lingual surface to the long axis of the tooth. The distances between the CEJ and CAB lines on the buccal and palatal/lingual surfaces were measured (Fig. 3).

### Statistical analysis

All data were analyzed using Statistical Package for Social Sciences (SPSS) 26.0 (software (IBM Corp., Armonk, NY, USA). The normality data was evaluated using Shapiro-Wilk’s test. Paired and unpaired t-tests were performed to compare the differences in measurements of alveolar bone changes for intra- and inter-group comparisons. Intra-class correlation coefficient (ICC) test through Cronbach’s Alpha was applied to measure the Intra- and inter-observer agreement. The statistically significant level was set as P < 0.05.

## Result

A total of one hundred thirty-three molars’ teeth roots of 24 patients met the inclusion criteria with a mean age of 31.1 + 9.9 years. The patients were assigned to two groups; the intrusion group (48.9%, n = 65 molars’ root) and the extrusion group (51.1%, n = 68 molars’ root). A good to excellent intra- and inter-examiner reliability was found, which was as low as 0.82 for the lingual surface of mandibular 2nd molar to as high as 0.99 for the lingual surface of maxillary 1st molar measurements. The amount of intrusion in the maxillary first and second molars was 1 ± 0.3 and 1.2 ± 0.5 mm, and in the mandibular ones was 0.9 ± 0.4 and 1.3 ± 0.4 mm, respectively. It is worthy also of mentioning that the amount of extrusion in the maxillary first and second molars was 0.9 ± 0.3 and 1.4 ± 0.5 mm, and in the mandibular ones, was 0.8 ± 0.2 and 1 ± 0.2 mm, respectively.

The positive values indicated bone deposition and negative values were used for bone resorption in the vertical plane. Table 1 presented the mean distances between the CEJ and the buccal and lingual alveolar bone crest (T0 and T1) and the mean changes (T0-T1) following treatment in the molars’ extrusion group. It showed a significant reduction of the alveolar bone changes for the mandibular right 1st molar (-1.05 ± 0.97 mm) and mandibular left 1st molar (-0.76 ± 1.12 mm), while all other sites changed insignificantly.

The mean distances between the CEJ and the buccal and lingual alveolar bone crest (T0 and T1) and the mean changes (T0-T1) following the molars’ intrusion group treatment are presented in Table 2. The buccal alveolar bone changes for the maxillary left 2nd molar and the lingual alveolar bone for the mandibular left 1st molar showed a significant reduction of the alveolar bone changes by -0.42 ± 0.77 mm, -0.64 ± 0.76 mm, respectively.

**Table 1:** Comparison of the mean difference of alveolar bone changes after extrusion movement of the maxillary and mandibular 1st and 2nd molars

Variables	Mean ± standard deviation SD (mm)			P value
	**T0**	**T1**	**Change T0-T1**	
**Buccal surface**				
Maxillary right 1st molar	1.95 ± 0.86	1.54 ± 0.80	0.41 ± 0.53	0.12
Maxillary right 2nd molar	2.60 ± 0.94	3.14 ± 1.25	-0.54 ± 0.70	0.22
Maxillary left 1st molar	1.44 ± 0.16	2.27 ± 0.66	-0.83 ± 0.79	0.21
Mandibular right 1st molar	2.68 ± 1.86	3.73 ± 2.03	-1.05 ± 0.97	0.01*
Mandibular right 2nd molar	1.91 ± 0.88	1.92 ± 1.28	-0.01 ± 0.87	0.95
Mandibular left 1st molar	1.43 ± 0.74	2.20 ± 1.27	-0.76 ± 1.12	0.04*
Mandibular left 2nd molar	1.64 ± 0.77	1.72 ± 0.46	-0.09 ± 1.29	0.78
**Lingual surface**				
Maxillary right 1st molar	2.54 ± 1.08	2.68 ± 1.42	-0.14 ± 1.45	0.82
Maxillary right 2nd molar	3.21 ± 0.67	3.99 ± 1.53	-0.78 ± 1.48	0.37
Maxillary left 1st molar	1.44 ± 0.85	2.96 ± 2.12	-1.53 ± 1.30	0.17
Mandibular right 1st molar	1.17 ± 0.66	1.18 ± 0.78	-0.01 ± 0.42	0.94
Mandibular right 2nd molar	0.98 ± 0.79	0.95 ± 0.73	0.03 ± 0.44	0.80
Mandibular left 1st molar	1.13 ± 0.99	1.00 ± 0.95	0.12 ± 0.62	0.52
Mandibular left 2nd molar	1.06 ± 0.89	1.16 ± 1.17	-0.09 ± 0.64	0.53

Notes: *(p ≤ 0.05), **(p ≤ 0.001), ***(p ≤ 0.000)

**Table 2:** Comparison of the mean difference of alveolar bone changes after intrusion movement of the maxillary and mandibular 1st and 2nd molars

Variables	Mean ± standard deviation SD (mm)			P value
	**T0**	**T1**	**Change T0-T1**	
**Buccal surface**				
Maxillary right 1st molar	1.66 ± 0.96	1.70 ± 1.35	-0.03 ± 0.85	0.90
Maxillary right 2nd molar	2.56 ± 3.50	1.80 ± 1.01	0.76 ± 3.55	0.49
Maxillary left 1st molar	1.98 ± 1.15	1.79 ± 1.42	0.19 ± 0.67	0.27
Maxillary left 2nd molar	1.37 ± 0.92	1.80 ± 1.09	-0.42 ± 0.77	0.03*
Mandibular right 1st molar	1.77 ± 0.64	2.86 ± 1.75	-1.08 ± 1.48	0.05
Mandibular right 2nd molar	0.97 ± 0.95	1.34 ± 0.59	-0.37 ± 0.61	0.40
Mandibular left 1st molar	2.03 ± 1.72	2.42 ± 1.80	-0.38 ± 0.65	0.14
Mandibular left 2nd molar	0.79 ± 0.21	0.69 ± 0.98	0.1 ± 0.76	0.88
**Lingual surface**				
Maxillary right 1st molar	2.57 ± 0.95	2.61 ± 1.30	-0.04 ± 0.63	0.83
Maxillary right 2nd molar	2.28 ± 1.09	2.51 ± 1.27	-0.23 ± 0.70	0.28
Maxillary left 1st molar	2.30 ± 1.08	2.30 ± 1.36	0.00 ± 0.656	0.99
Maxillary left 2nd molar	2.02 ± 1.32	2.17 ± 1.24	-0.14 ± 0.80	0.45
Mandibular right 1st molar	1.23 ± 1.33	1.53 ± 1.08	-0.30 ± 0.43	0.07
Mandibular right 2nd molar	0.28 ± 0.17	0.81 ± 0.52	-0.53 ± 0.47	0.19
Mandibular left 1st molar	1.16 ± 0.78	1.80 ± 1.26	-0.64 ± 0.76	0.04*
Mandibular left 2nd molar	0.48 ± 0.24	0.82 ± 1.16	-0.34 ± 0.91	0.69
Notes: *(p ≤ 0.05), **(p ≤ 0.001), ***(p ≤ 0.000)				

Table 3 shows the average differences in pre- and post-treatment values (T0 -T1) between the maxillary and mandibular groups following treatment in the molars’ extrusion and intrusion groups. The extrusion group showed a significant treatment difference in the buccal alveolar bone changes for the mandibular right 1st molar (-0.55 ± 0.87 mm), and the maxillary left 2nd molar (-0.42 ± 0.77 mm) in the intrusion group.

The average differences between pre- and post-treatment values (T0 -T1) of the buccal and lingual alveolar bone changes following treatment in the molars’ extrusion and intrusion group are presented in Table 4. It showed a significant treatment difference in the extrusion group in the buccal alveolar bone for the maxillary left 1st molar (-1.28 ± 0.04 mm), while there were insignificant differences in all other sites.

**Table 3:** Comparison of the mean difference of alveolar bone changes between the maxillary and mandibular 1st and 2nd molars following extrusion and intrusion movements

Variable	Mean ± standard deviation SD (mm)							
	**Right 1st molar**		**Right 2nd molar**		**Left 1st molar**		**Left 2nd molar**	
	**Max**	**Mand**	**Max**	**Mand**	**Max**	**Mand**	**Max**	**Mand**
**Extrusion**								
**Buccal**	0.17 ± 0.37	-0.55 ± 0.87	-0.42 ± 1.33	-0.01 ± 0.78	-0.13 ± 0.41	-0.49 ± 0.96	NA	NA
**P value**	0.003 ** *		0.27		0.160		--	
**Lingual**	0.05 ± 0.90	-0.01 ± 0.30	-0.26 ± 0.79	0.02 ± 0.40	-0.25 ± 0.73	0.08 ± 0.49	NA	NA
**P value**	0.79		0.18		0.12		--	
**Intrusion**								
**Buccal**	-0.08 ± 0.70	-0.57 ± 1.18	0.61 ± 2.79	-0.06 ± 0.26	0.16 ± 0.63	-0.18 ± 0.47	-0.42 ± 0.77	0.01 ± 0.19
**P value**	0.14		0.32		0.075		0.03 *	
**Lingual**	-0.09 ± 0.55	-0.16 ± 0.34	-0.04 ± 0.71	-0.09 ± 0.26	0.00 ± 0.61	-0.30 ± 0.60	-0.14 ± 0.80	-0.04 ± 0.25
**P value**	0.66		0.79		0.14		0.60	

Notes: *(p ≤ 0.05), **(p ≤ 0.001), ***(p ≤ 0.000); NA, none available

**Table 4:** Comparison of the average difference of alveolar bone changes between extrusion and intrusion movements

Variables	Mean ± standard deviation SD (mm)		P value
	**Extrusion**	**Intrusion**	
**Buccal surface**			
Maxillary right 1st molar	0.14 ± 0.30	-0.09 ± 0.75	0.29
Maxillary right 2nd molar	-0.52 ± 1.46	0.8 ± 2.98	0.16
Maxillary left 1st molar	-1.28 ± 0.04	0 ± 0	0.01*
Maxillary left 2nd molar	NA	NA	NA
Mandibular right 1st molar	-0.44 ± 0.83	-0.22 ± 0.70	0.41
Mandibular right 2nd molar	0.06 ± 0.61	-0.01 ± 0.15	0.60
Mandibular left 1st molar	-0.34 ± 0.83	-0.07 ± 0.36	0.15
Mandibular left 2nd molar	-0.05 ± 0.80	0.02 ± 0.22	0.77
**Lingual surface**			
Maxillary right 1st molar	-0.06 ± 0.84	-0.10 ± 0.59	0.89
Maxillary right 2nd molar	-0.32 ± 0.86	0.08 ± 0.59	0.22
Maxillary left 1st molar	-2.19 ± 0.87	0.00 ± 0.00	0.17
Maxillary left 2nd molar	NA	NA	NA
Mandibular right 1st molar	0.01 ± 0.27	-0.10 ± 0.29	0.25
Mandibular right 2nd molar	0.02 ± 0.35	-0.07 ± 0.24	0.31
Mandibular left 1st molar	0.05 ± 0.41	-0.19 ± 0.52	0.07
Mandibular left 2nd molar	-0.12 ± 0.65	-0.05 ± 0.29	0.73

Notes: *(p ≤ 0.05), **(p ≤ 0.001), ***(p ≤ 0.000); NA, none available

## Discussion

The primary aims of orthodontic treatment are to optimize desired tooth movement while minimizing adverse effects on the periodontal structure surrounding the teeth. In orthodontic treatment, considering alveolar bone support for optimal stability of the teeth and periodontal health is important [1]. The relationship between orthodontic treatment and alterations in the distance between the CEJ and the bone crest has received significant attention in the literature [20] however, variations in orthodontic techniques and evaluation criteria used in the studies have limited the comparison of results [17].

CBCT is used to investigate alveolar bone levels because it can evaluate more tooth surfaces than conventional radiography [19]. Based on available evidence, clear aligners can serve as a suitable alternative to traditional fixed orthodontic treatment in the non-extraction treatment of mild to moderate malocclusions in non-growing patients [5, 21]. Previous studies have evaluated treatment outcomes of clear Aligners and their biomechanical mechanisms, but the exerted side effects remain unknown [22, 23].

This study found a statistically significant vertical bone loss in patients with extrusion movement, particularly in the buccal surface of the mandibular right 1st molars and mandibular left 1st molars. A study conducted by Lund et al. [19] found that loss in the vertical bone changes with a distance of > 2 mm was found in 2% (mesial surface of mandibular 1st molar) to 16% (distal surface of maxillary 1st molar). Other studies with similar results revealed that the distance from the cementoenamel junction to the bone crest changed after orthodontic treatment; the distance was greater than 2 mm in 19% after treatment representing the loss of the alveolar bone height [17]. Although another study showed a loss in the vertical bone height with a mean distance of 2.4 mm on the buccal and lingual surfaces of mandibular teeth after orthodontic treatment, the authors of these studies concluded that the morphology of the alveolar bone is a limiting factor for orthodontic movement; these limiting factors include bone morphology, magnitude and direction of applied force and the detriment of periodontal injury [24]. Additionally, the previous study reported a significant loss in the buccal alveolar bone height at the central incisors and 1st molar [25]. Garlock et al. [26] reported on average 1.12 mm of buccal bone recession at the mandibular central incisor, with high variability after non-extraction treatment with a self-ligating appliance. Other factors need to be considered, such as how orthodontic teeth movement will produce a biological reaction that can be different in adults than children.

In the intrusion group, our result showed an increase in mean distance from the CEJ to the alveolar bone crest in the buccal surface of the maxillary left 2nd molars (-0.42 ± 0.77, P = 0.03) and in the lingual surface of the mandibular left 1st molar (-0.64 ± 0.76, P = 0.04) representing negative alveolar bone changes. This result had an agreement with a study conducted by Atik et al. [1] who evaluated the changes in the maxillary alveolar bone height after incisor intrusion using conventional orthodontic treatment, and showed the percentage of the loss of the alveolar bone height on the labial side of the maxillary right and left incisors were significantly lower in the mini-screw group than in the base-arch group. This disagreed with the study by Guo et al. [14] that the loss percentage of alveolar bone height on the lingual side was more significant than that of the labial side.

Tooth movements that shift the teeth away from the alveolar ridge may play a crucial role in causing bone dehiscence (a type of jawbone defect) [1]. In the current study, all surrounding alveolar bone near the molars was subjected to an inward force with a backward direction, which might lead to a concentration of stress and deformation on the buccal alveolar crest which was in line with the study conducted by Bimstein [27]. A survey conducted by Son et al. [28] hypothesized that intrusion of incisors might compensate for vertical palatal bone loss during maxillary incisors intrusion and retraction. However, the findings of their research did not support that hypothesis. Therefore, the loss of palatal bone must be closely monitored during the treatment.

Regarding the average differences in pre- and post-treatment values between the maxillary and mandibular groups, our results showed a significant difference in the buccal alveolar bone changes for the mandibular right 1st molar in the extrusion group, and the maxillary left 2nd molar in the intrusion group. Also, average differences in pre- and post-treatment values of the buccal and lingual/palatal alveolar bone changes between extrusion and intrusion groups showed a significant treatment change in the extrusion group in the buccal alveolar bone for the maxillary left 1st molar. It appears that this finding has not been observed in other reports. On the other hand, Miyama et al. [29] reported a decrease in the distance between CEJ and alveolar bone crest after intrusion or extrusion. In contrast, Castro et al. [17] reported that the distance between CEJ and the crest of the alveolar bone did not change by orthodontic movement.

Some limitations of this study have to be mentioned, including the immediate post-treatment analysis and lack of follow-up images. Therefore, we cannot state whether the defects undergo spontaneous resolution over time or not. Furthermore, we could not evaluate gender differences due to the unequal number of males and females in the study sample. There might be differences in hormonal changes between males and females and between different age groups, which may also affect bone remodeling during orthodontic tooth movement. Future clinical studies are recommended to evaluate the treatment changes in all teeth and surfaces and examine the effect of the different malocclusions, especially the vertical facial patterns, possibly different bone morphology, on bony changes during orthodontic treatment of both genders using Clear Aligners. Finally, this study evaluated short-term alveolar bone changes (immediately postoperative), and further studies evaluated the long-term changes, including the possible determination of whether these changes occurred due to molars’ vertical movements or actual bone resorption or deposition.

The null hypothesis was rejected confirming that there is a difference in the alveolar bone changes following maxillary and mandibular molars’ intrusion and extrusion movements following clear aligners; this difference is in the form of a reduction of the alveolar bone changes around molars.

## Conclusion

- The buccal alveolar bone changes is considered the most affected surface following maxillary and mandibular molars’ intrusion and extrusion movements using clear aligners, with mandibular molars being more affected than the maxillary ones.
- The extrusion movement showed a significant negative in alveolar bone changes at the buccal surface of the mandibular right and left 1st molars.
- In intrusion movement; the maxillary left 2nd molar and mandibular left 1st molar were the most affected teeth on the buccal and lingual sides, respectively.
- The mandibular right 1st molar in the extrusion and the maxillary left 2nd molar in the intrusion movements showed a significant negative in the buccal surface when comparing both arches.

## Data Availability

All data generated or analyzed during this study are included in this published article.

## Abbreviations

ICC: Intra-class correlation coefficient
T0: before treatment
T1: after treatment
CBCT: Cone Beam Computed Tomography
CEJ: Cementoenamel junction
DICOM: Digital Imaging and Communication in Medicine

## References

[1] Atik E, Gorucu-Coskuner H, Akarsu-Guven B, Taner T (2018). Evaluation of changes in the maxillary alveolar bone after incisor intrusion. korean J Orthod.

[2] Yousif A, Elmarhoumy SM (2020). Alveolar bone changes after orthodontic tooth movements: a CBCT Study. Egypt Dent J.

[3] Graber Lee W, Vanarsdall Robert L Jr, Vig Katherine WL. Orthodontics:current principles & techniques. 2012.

[4] Sun Q, Lu W, Zhang Y, Peng L, Chen S, Han B (2021). Morphological changes of the anterior alveolar bone due to retraction of anterior teeth: a retrospective study. Head Face Med.

[5] Jiang T, Wang JK, Jiang YY, Hu Z, Tang GH (2021). How well do integrated 3D models predict alveolar defects after treatment with clear aligners?. Angle Orthod.

[6] Wehrbein H, Bauer W, Diedrich P (1996). Mandibular incisors, alveolar bone, and symphysis afterorthodontic treatment. A retrospective study. Am J Orthod Dentofac Orthop.

[7] Southard KA, Liu WJ, Behrents RG. Extraction versus periodontal-orthodontic treatment: a case report. Quintessence Int (Berl). 1991;22. 1882054

[8] Moshiri M. Product review and demonstration of the Invisalign clear aligner system. 2021.

[9] Womack WR, Ahn JH, Ammari Z, Castillo A (2002). A new approach to correction of crowding. Am J Orthod Dentofac Orthop.

[10] Phan X, Ling PH. Clinical limitations of Invisalign. J Can Dent Assoc (Tor). 2007;73. 17439714

[11] ZACHRISSON BU (1973). Periodontal condition in orthodontically treated and untreated individuals I. loss of attachment, gingival pocket depth and clinical crown height. Angle Orthod.

[12] Janson G, Bombonatti R, Brandão AG, Henriques JFC, de Freitas MR (2003). Comparative radiographic evaluation of the alveolar bone crest after orthodontic treatment. Am J Orthod Dentofac Orthop.

[13] Tsao DH, Kazanoglu A, McCasland JP (1983). Measurability of radiographic images. Am J Orthod.

[14] Guo Q, Zhang S, Liu H, Wang C, Wei F, Lv T (2011). Three-dimensional evaluation of upper anterior alveolar bone dehiscence after incisor retraction and intrusion in adult patients with bimaxillary protrusion malocclusion. J Zhejiang Univ Sci B.

[15] ZHOU D. Comparison of alveolar bone changes in maxillary anterior area secondary to different kinds of retraction method of anterior teeth: a cone-beam computed tomography study. J Shanghai Jiaotong Univ (Medical Science). 2018;:1375–80.

[16] Singh G. Textbook of orthodontics. 2007.

[17] Castro LO, Castro IO, de Alencar AHG, Valladares-Neto J, Estrela C (2016). Cone beam computed tomography evaluation of distance from cementoenamel junction to alveolar crest before and after nonextraction orthodontic treatment. Angle Orthod.

[18] Ma J, Huang J, Jiang J (2019). Morphological analysis of the alveolar bone of the anterior teeth in severe high-angle skeletal class II and Class III malocclusions assessed with cone-beam computed tomography. PLoS ONE.

[19] Lund H, Gröndahl K, Gröndahl H (2012). Cone beam computed tomography evaluations of marginal alveolar bone before and after orthodontic treatment combined with premolar extractions. Eur J Oral Sci.

[20] Guo R, Zhang L, Hu M, Huang Y, Li W (2021). Alveolar bone changes in maxillary and mandibular anterior teeth during orthodontic treatment: a systematic review and meta-analysis. Orthod Craniofac Res.

[21] Papadimitriou A, Mousoulea S, Gkantidis N, Kloukos D (2018). Clinical effectiveness of Invisalign® orthodontic treatment: a systematic review. Prog Orthod.

[22] Zheng M, Liu R, Ni Z, Yu Z (2017). Efficiency, effectiveness and treatment stability of clear aligners: a systematic review and meta-analysis. Orthod Craniofac Res.

[23] Liu Y, Hu W (2018). Force changes associated with different intrusion strategies for deep-bite correction by clear aligners. Angle Orthod.

[24] Garib DG, Henriques JFC, Janson G, Freitas MR, Coelho RA (2005). Rapid maxillary expansion—tooth tissue-borne versus tooth-borne expanders: a computed tomography evaluation of dentoskeletal effects. Angle Orthod.

[25] Morais JF, Melsen B, de Freitas KMS, Castello Branco N, Garib DG, Cattaneo PM (2018). Evaluation of maxillary buccal alveolar bone before and after orthodontic alignment without extractions: a cone beam computed tomographic study. Angle Orthod.

[26] Garlock DT, Buschang PH, Araujo EA, Behrents RG, Kim KB (2016). Evaluation of marginal alveolar bone in the anterior mandible with pretreatment and posttreatment computed tomography in nonextraction patients. Am J Orthod Dentofac Orthop.

[27] Bimstein E, Crevoisier RA, King DL (1990). Changes in the morphology of the buccal alveolar bone of protruded manidbular permanent incisors secondary to orthodontic alignment. Am J Orthod Dentofac Orthop.

[28] Son EJ, Kim SJ, Hong C, Chan V, Sim HY, Ji S (2020). A study on the morphologic change of palatal alveolar bone shape after intrusion and retraction of maxillary incisors. Sci Rep.

[29] Miyama W, Uchida Y, Motoyoshi M, Motozawa K, Kato M, Shimizu N (2018). Cone-beam computed tomographic evaluation of changes in maxillary alveolar bone after orthodontic treatment. J Oral Sci.

